# Acceptability of a Hypothetical Zika Vaccine among Women from Colombia and Spain Exposed to ZIKV: A Qualitative Study

**DOI:** 10.3390/vaccines8040580

**Published:** 2020-10-03

**Authors:** Elena Marbán-Castro, Ana Villén-Gonzalvo, Cristina Enguita-Fernàndez, Kelly Carolina Romero-Acosta, Anna Marín-Cos, Germán J. Arrieta, Salim Mattar, Clara Menéndez, Maria Maixenchs, Azucena Bardají

**Affiliations:** 1 ISGlobal, Hospital Clínic-Universitat de Barcelona, 08036 Barcelona, Spain; ana.villen.gonzalvo@gmail.com (A.V.-G.); cristina.enguita@isglobal.org (C.E.-F.); annamarin1986@gmail.com (A.M.-C.); clara.menendez@isglobal.org (C.M.); maria.maixenchs@isglobal.org (M.M.); azucena.bardaji@isglobal.org (A.B.); 2 Consorcio de Investigación Biomédica en Red de Epidemiología y Salud Pública (CIBERESP), 28029 Madrid, Spain; 3 Faculty of Humanities and Education, Corporación Universitaria del Caribe (CECAR), Sincelejo, Sucre 700001, Colombia; kelly.romero@cecar.edu.co (K.C.R.-A.); GERMAN.ARRIETA@cecar.edu.co (G.J.A.); 4 Clínica Salud Social, Sincelejo, Sucre 700001, Colombia; smattar@correo.unicordoba.edu.co; 5 Instituto de Investigaciones Biológicas del Trópico, Universidad de Córdoba, Montería 230002, Córdoba, Colombia; 6 Centro de Investigação em Saúde de Manhiça (CISM), Maputo 1929, Mozambique

**Keywords:** Zika, vaccine, acceptability, pregnancy, qualitative, grounded theory

## Abstract

Zika virus (ZIKV) can cause pregnancy loss and congenital Zika syndrome, among other poor health outcomes. The ZIKV epidemic in 2015–2017 disproportionately affected pregnant women in poor-resource settings. We aimed to understand perceptions and attitudes towards a hypothetical ZIKV vaccine, women’s willingness to be vaccinated, and potential barriers and facilitators for vaccine acceptance in 1) migrant women living in Spain who travelled to their countries of origin and were diagnosed with ZIKV infection during pregnancy, and their healthcare providers, and 2) women living in Colombia who delivered a child with microcephaly. An exploratory qualitative study based on phenomenology and grounded theory was conducted. Data were collected through in-depth, paired and semi-structured interviews. Overall, women from both sites were willing to receive a hypothetical ZIKV vaccine. However, some expressed concerns of being vaccinated during pregnancy, yet they would accept it if the vaccine was recommended by a healthcare professional they trust. Main fears towards vaccination were related to vaccine safety and potential adverse effects on child’s health. Women reported feeling hesitant to participate in a ZIKV vaccine trial. These results may contribute to guiding the effective delivery of future ZIKV vaccines among populations most at risk and particularly vulnerable.

## 1. Introduction

The Zika virus (ZIKV) epidemic spread worldwide during 2015 and 2017, disproportionately affecting populations living in countries in Central and South America [1,2]. ZIKV usually causes a mild disease in immunocompetent adults, but Zika infection during pregnancy has been associated with spontaneous abortions, stillbirths, and a wide range of severe neurological congenital anomalies in fetuses and infants, known as congenital Zika syndrome (CZS) [3]. The consequences of ZIKV in uterus’ exposure can be prenatally or postnatally diagnosed, over a child´s first years of life [4,5]. The whole spectrum of the consequences of the epidemics cannot be explained just from a clinical or epidemiological view. In turn, interdisciplinarity is essential to understand the toll of carrying a child with ZIKV-related disabilities; the mental health of populations affected by ZIKV, especially of caregivers of children with disabilities, suffering during pregnancy for uncertainties regarding to child health; socio-economic impoverishment due to special care needs; and ZIKV-associated stigma [6,7]. Studies addressing the socio-economic impact of the ZIKV infection among affected families are scarce. Social, economic and cultural consequences of this emerging epidemic remain largely unknown; and its impact magnitude represents a challenge to be assessed in the long-term [7]. Recent outbreaks such as Ebola, Zika and cholera have raised the need to understand the social pathways of disease transmission and barriers to health seeking behavior [8,9,10]. The role of anthropology in confronting emerging outbreaks is pivotal for integration of sociocultural approaches in response to international health crises [8,11,12].

Previous research has found gaps in ZIKV knowledge regarding preventive measures and vaccine acceptability [7,13,14,15]. Different risk perceptions among patients and healthcare professionals might influence pregnant women attitudes towards proposed measures for controlling the disease, e.g., repellent use while traveling to endemic areas and condom use throughout pregnancy [7]. This explains the need to understand women´s health seeking behavior, their response and prevention practices before and after communication with healthcare professionals, and their reproductive decisions. Currently, there is no specific treatment for ZIKV infection, thus, given the severity of the disease and challenges in preventing vector-borne infections, the most effective tool for protecting women from the dramatic consequences of ZIKV would be vaccination. ZIKV vaccine research is currently undergoing phase I and phase II clinical trials with different candidates under development [16,17,18,19].

Vaccination has demonstrated to be one of the most cost-effective health interventions to avoid disease-associated morbidity and mortality, but there are hesitant groups about the clinical development and regulatory process of vaccines [20]. Vaccine hesitancy refers to a situation where people are doubtful about vaccinations or where they choose to refuse immunizations despite the availability of vaccination services. Lack of knowledge about vaccines and concerns about vaccine safety are identified as the main causes of vaccine hesitancy, a problem already recognized by the WHO as a major public health threat for humanity [21]. Extreme media attention, as received by the ZIKV epidemic, can boost public concern about both the disease and protective strategies, such as vaccines. However, it may also increase people’s likelihood to refuse a ZIKV vaccine once it will become available. This highlights the importance of exploring people’s perceptions and concerns regarding ZIKV disease preventive strategies through a qualitative research methodology [22].

Few studies have addressed the acceptability of a hypothetical ZIKV vaccine should it become available in the near future [15,23,24,25,26,27,28,29,30,31,32], and their geographic distribution is not representative of the populations at risk. The majority of studies were performed in the USA, four in South East Asia, and only two in South America, in Brazil and Guatemala. To date, no studies have addressed acceptability of a hypothetical ZIKV vaccine in a European context among travelers or migrants. Considering the potential of ZIKV to become a global threat again in the future, and given its dramatic consequences on the health of women and children, it is crucial to understand the level of knowledge, perceived benefits, drawbacks, concerns and expectations of a ZIKV vaccine among women potentially exposed to the virus living in or traveling to endemic areas, their partners and healthcare professionals.

This study aimed to understand perceptions, views and attitudes towards ZIKV vaccines to prevent the effects of the infection in pregnancy and infants among women exposed to ZIKV during pregnancy and healthcare professionals, as well as the willingness of women to receive the vaccine if one was developed and available. Though the purpose of the study was not to compare different populations, the views about a hypothetical ZIKV vaccine in two different contexts are described: (1) migrant women from Central and South America living in Spain who travelled to their countries of origin and were diagnosed with ZIKV infection during pregnancy, and healthcare professionals who looked after these women; (2) women living in Colombia who delivered a baby with microcephaly during the ZIKV epidemic.

## 2. Materials and Methods 

### 2.1. Study Sites and Population

The study took place in two different contexts and involved two population groups. In Spain, the study was conducted between September 2018 and February 2020. Participants were identified from a surveillance study of ZIKV during pregnancy conducted at the Department of Maternal-Fetal Medicine, BCNatal-Barcelona Center of Maternal-Fetal and Neonatal Medicine, Hospital Clínic (HCB) and Hospital Sant Joan de Déu (HSJD). Study participants were women who had been diagnosed with ZIKV infection (confirmed or probable cases) during pregnancy, and a subset of healthcare professionals who assisted them during pregnancy and childcare [7]. In Colombia, the study was performed between April and July 2019. Participants were identified from a study conducted on infants with microcephaly in rural and peri-urban areas in Córdoba and Sucre Departments, born during the ZIKV epidemic at the Hospital “Clínica Salud Social”, in Sincelejo, Sucre [32,33]. Mothers of infants with microcephaly were invited to participate in the study, regardless reporting ZIKV-related symptoms during pregnancy.

Inclusion criteria for study participants in Spain were defined as: having travelled to an endemic area for ZIKV during pregnancy; attending antenatal care at HCB; and willing to take part in study procedures. Inclusion criteria for healthcare professionals were defined as: having provided clinical or preventive care to pregnant women who had travelled to ZIKV endemic areas and/or to their children of up to two years of age. Invited healthcare providers included both obstetricians, pediatricians, and tropical diseases’ specialists. Inclusion criteria for study participants in Colombia were defined as: having lived in a ZIKV endemic area, having given birth a newborn diagnosed with microcephaly during the ZIKV epidemic or being his/her primary caregiver, and willing to be interviewed and audio-recorded as part of study procedures.

A minimum sample size of 15 participants among both sites was defined based on previous studies’ experience in reaching the saturation point and theoretical saturation. The saturation point is reached after several interviews do not lead to new concepts [34,35,36,37]. Purposive and snowball sampling was used to enroll participants. Within this sample, women of different backgrounds, ages, nationalities, and experiences were included in the study. The inclusion of women from these two different contexts provides extensive information. Interviewed women in Spain had healthy children in most cases, while those in Colombia had children born with microcephaly during the ZIKV epidemic. In both groups, perceptions about a hypothetical ZIKV vaccine were discussed from a theoretical point of view. Purposive sampling was applied to include healthcare professionals who had provided healthcare to study participants in Spain and their children.

### 2.2. Study Design

This was an exploratory qualitative study based on phenomenology and grounded theory. Grounded theory is an inductive approach where theoretical generalizations emerge from the data rather than being assumed beforehand [36]. Phenomenology is an approach to understand first-hand experiences of those involved in a phenomenon of interest, and it is particularly useful to examine complex and sensitive topics [37].

### 2.3. Data Collection

In Spain, data were collected through in-depth Interviews (IDI) and paired interviews (PI) [35,37,38] for women exposed to ZIKV during pregnancy (confirmed and/or suspected cases), and semi-structured interviews (SSI) for healthcare professionals. Data were collected between December 2018 to February 2020. In Colombia, data were collected through IDI with primary caregivers (mothers) of children born with microcephaly. Data collection took place from April 2019 to September 2019. In both countries, interviews were carried out at the place of preference of the participants, including health facilities, participant´s place of residence or public places. PIs took place in a room at the Maternity of HCB. All interviews were digitally recorded, and notes were taken. Researchers came together regularly to discuss key findings, difficulties and any appropriate change to the guides, according the data that emerged. All interviews were transcribed, anonymized, and data were double transcribed and coded for quality control purposes.

### 2.4. Data Analysis

Grounded theory was used as a methodological and analytical approach. Research began with no pre-existing hypothesis, which allowed a theory to inductively emerge from the data, following systematic and circular data collection and analysis. Theory generation was based on comparative analyses among data collected from different participants, and pre-existing conceptualizations were not used [37]. Data analysis was conducted by two researchers and validated by one experienced co-investigator. Transcripts and notes were imported into Dedoose^®^ software (SocioCultural Research Consultants, LLC, Manhattan Beach, CA, USA) and thoroughly read, coded, organized and categorized. Codes were generated as data were gathered, refined, added or deleted according to their relevance based on research questions, already coded data, and emergent data. From codes, themes were constructed, discussions were built around each theme and subtheme, and from investigators’ discussions, main conclusions emerged. Our study complies with the Consolidated Criteria for Reporting Qualitative Research (COREQ) checklist [39].

### 2.5. Ethical Considerations

Ethical approval for the study was granted by the Ethics Review Committee of the Hospital Clínic of Barcelona, Spain (CEIC) (Reg. No. HCB/2016/0250), by the Ethics Committee of the Universidad de Córdoba, Montería, Colombia (Reg. No. FMVZ-001-2016), and Clínica Salud Social, Sincelejo, Colombia, (Reg. No. F-GI-IV-001). The study was conducted in accordance with the Good Clinical Practice Guidelines, and under the provisions of the Declaration of Helsinki and local rules and regulations. Participants gave written and oral consent for interviews to take place and audio recording. All names in the transcripts were deleted to guarantee subject anonymity.

## 3. Results

### 3.1. Study Profile

In Spain, seventeen women with confirmed or probable ZIKV infection during pregnancy were enrolled in the study; twelve women in the IDIs, and five women in the PIs. The average age was 31 years old (range from 22 to 42 years old). Countries of origin of study women included the Dominican Republic (6/17), and Honduras (5/17); there were two participants from Colombia, two from Venezuela, one from Brazil, and one from Guatemala. All the participants had completed (at least) primary education, six of them reported having studied at the university, and nearly half of them reported working at home (housewife) (10/17). Ten out of the seventeen women declared to be Christians. The average time living in Spain was 8.4 years, with a minimum period of 1 year and a maximum of 18 years. Seven healthcare professionals participated in the SSIs: three obstetricians, two pediatricians, and one tropical medicine and international health specialist. All of them were women.

In Colombia, seven women who had delivered an infant with microcephaly were enrolled into the study. In addition, one grandmother and main caregiver of a child with microcephaly was also enrolled. In Colombia, the mean age of study participants was 26 years (range from 18 to 43 years). Six out of eight women declared to be Christians. All of them were from the lowest socioeconomic status in Colombia according to the Central Government classification defined by households’ characteristics [40]. Table 1 describes the sociodemographic characteristics of women participating in the study.

### 3.2. General Perceptions about a ZIKV Vaccine

Women were asked about their perceptions regarding ZIKV vaccines and about their willingness to receive a hypothetical ZIKV vaccine if one was developed and available in the future. All participants were aware of the preventative purposes of vaccines, and recognized its utility to avoid disease.

“*I think that all [vaccines] exist to prevent that, if a disease arises to your body… if it hits you, it will not be so hard or at least, it is not going to kill you, because a lot of people died before due to diseases that are now being treated. I think that they… I think they are necessary because today there are a lot of viruses, too many diseases and we have the capacity to obtain help in that sense, because before there was not medical attention for those diseases that are now being treated.*” (Sincelejo, Sucre, 23 years old)

One of the participants in a PI in Spain mentioned that in their countries of origin, they did not have “*anti-vaccine groups like in Europe*”. Overall, all women in both sites saw the existence of a ZIKV vaccine as a very positive announcement. In Spain, women declared their main reason to get vaccinated would be to avoid the disease in their children and to avoid psychological stress and poor emotional status experienced during their pregnancy due to the uncertainty of having the disease and the consequences it would have on them and their babies. In Colombia, women reinforced the idea of avoiding living this experience again, the uncertainty of duration of protection (antibodies), and efforts to decrease the number of children born with congenital Zika syndrome “*So there are not so many children [with Congenital Zika Syndrome]… because children suffer, but also their parents*” as reasons to be vaccinated.

Women in Spain considered that a vaccine would give them “*peace of mind*” and a “*sense of security*”. Women in both sites expressed that everybody should be vaccinated, not only pregnant women. Reasons for this statement included protection by herd immunity; because ZIKV can be transmitted by sexual contact; and because “*mosquitos are everywhere*”. Participants in Colombia agreed that vaccines need to be universal for the same reasons. Additionally, one woman argued that, in fact, ZIKV can cause severe disease in the elderly.

Healthcare professionals also showed their interest in a ZIKV vaccine to be developed and administered to those potentially exposed to the virus. They expressed their thoughts about this vaccine to be well received by populations at risk (those living in endemic areas or those traveling to those places). Interviewed healthcare professionals commented about who should be vaccinated, when a vaccine is available.

“*Well, I wish this vaccine to appear… I think it would be… as long as it does not exist… the infection in our setting, due to the vector… I would recommend within our setting, to non-immunized patients who… well, who travelled, and had to travel to these settings, as it is for Yellow Fever. And in places where infection is endemic, to everybody, or at least to all the female population before pregnancy, girls and adolescents.*” (Healthcare professional, number 1)

“*I think that in endemic areas, it would be a vaccine to be administered during women’s adolescence, depends on the duration of vaccine [immunity], that’s why I told you, depends on the type of the vaccine, and it would be well received [by the population] because it is going to avoid morbidity and mortality among fetuses.*” (Healthcare professional, number 4)

### 3.3. Route of Administration of a ZIKV Vaccine

In order to assess if the route of administration of the vaccine could be a factor to facilitate or to hinder acceptance of the vaccine, interviewees were asked about their preferences. Responses were diverse. Some women preferred tablets, and others droplets, because they were afraid of needles. Nevertheless, the majority of participants explained they preferred injected vaccines because they “*were considered more effective, strong and reliable*”, they “*went directly to blood*”, and they should be administered in one single shot at a health center so people cannot avoid or forget taking it.

“*[I prefer] something that reaches. It’s like, I say, ‘Because if you get an injection, then it reaches better because it goes more directly to blood’ isn’t it? […] I have panic to needles but I prefer it [the vaccine] to go directly*” (Honduras, 33 years old, 12 years in Spain)

Others did not express any concern or preference regarding the route of administration. The most important factor for them was vaccine safety and effectiveness to protect children’s health.

“*As long as it would be to… to control the disease, it doesn’t matter [it] to be drunk, to be injected, to be intravenously, wherever, but to be… something that can control [the virus] and that it did not get to that point… that stage in which a baby could be born sick.*” (The Dominican Republic, 42 years old, 18 years in Spain)

In Colombia, a woman explained that injected vaccines for adults were the most convenient, but orally would be a better route of administration if targeted to children in order to avoid the pain of the puncture.

### 3.4. Preferred Timing of Administration of a ZIKV Vaccine

Both in Colombia and Spain, women explained they would want to be vaccinated as soon as possible, before pregnancy, or in the first months of gestation, arguing that it would offer more protection against the negative effects of ZIKV on their fetuses. However, they also reinforced the idea of being vaccinated when their healthcare assistants recommend them to do so (first, second or third trimester of pregnancy).

“*Well, if it [the vaccine] could be [administered] before pregnancy, much better. But I don’t know… I think [I prefer] at the beginning of pregnancy, isn’t it? To avoid any… to prevent any future infection.*” (Honduras, 31 years old, 5 years in Spain)

Participants in Colombia explained the same reasoning for early vaccination (first trimester), or even the first month of pregnancy, as the elected one as a way to avoid maximum exposure and children’s infection, though they would follow recommendations from their healthcare professionals.

### 3.5. Willingness to Receive a ZIKV Vaccine

Almost all women expressed their willingness to be vaccinated with a hypothetical ZIKV vaccine, as long as it is recommended by their healthcare provider. The main reasons for accepting a hypothetical ZIKV vaccine are presented in Table 2.

Healthcare professionals explained that pregnant women usually are fearful about receiving vaccines during gestation, but their acceptability depends, according to healthcare professionals, on factors such as perception of risk, educational level and understanding of the benefits, and how information is provided from healthcare professionals to them.

### 3.6. Barriers to the Acceptance of a ZIKV Vaccine

In Spain, there were only two barriers identified by participants to accept vaccination, or circumstances in which women would hesitate before accepting. These would be to be pregnant or breastfeeding at the time of vaccine administration, due to fear and concerns about the safety of the vaccine and its impact on the health of the fetus and the baby. At that point, they would base their decision on the presence of ZIKV in their country of residence or plans of travel to a risk country with a current outbreak.

“*I think that babies take everything. So, we don’t know if the drug could hurt the baby or could be allergic… he/she can be allergic to something contained in the drug.*” (The Dominican Republic, 28 years old, 12 years in Spain)

In Colombia, barriers to accept vaccination included the decision, in some women, of not getting pregnant again to avoid the suffering related to ZIKV risk (consequently no need for a vaccine) and the vaccine not being included in the health system routine care or not being given for free. Only some participants declared they would accept ZIKV vaccination during breastfeeding. The vast majority of women, in both sites, explained their fears in “*passing ZIKV*” to their babies and “*possible adverse consequences in fetuses*” if the vaccine was administered during breastfeeding. In some cases, they said they would accept vaccination during breastfeeding only if recommended by a healthcare professional.

“*No, because… mmm… they say that when you are breastfeeding you cannot get any injection.*” (Sincelejo, Sucre, 23 years old)

Some women explained their concerns about possible adverse effects of the vaccine. A key determinant to accept vaccination would be to be sure that the vaccine would not cause any damage in their pregnancy or their children’s health “*if there are no risks*”, “*if it’s completely safe for the baby*” and “*as long as the fetus is OK*”.

“*I’ve seen that here [Spain], for example, babies’ vaccines, there are a lot of people that fear to put them a vaccine for secondary effects, but I think… well, in my opinion, vaccines are done to so, to prevent possible diseases, and well, if one can have it to be less worried, so, it’s perfect!… mmm secondary effects, well, none is exempt that that could happen but, it’s like… thinking in positive.*” (Venezuela, 25 years old, 1 year in Spain)

In Spain, the cost of the vaccine was not reported as a barrier to accept vaccination. Only a woman in Colombia explained that a barrier could be that the vaccine was not given for free. Another woman in Colombia explained her experience saving money for a vaccine that was not nationally covered in the immunization schedule for children “*even if we don’t eat in a month, but we’ll get the vaccine*”.

### 3.7. Hypothetical Acceptability to Participate in a Clinical Trial

Most women declared they would not like to participate in a clinical trial due to concerns about the effect of the vaccine on children´s health, possible adverse effects following immunization and fear and uncertainty about vaccine safety. Those women who declined to participate in a trial declared that they could reconsider it in order to help other women not to go through their same situation.

“*No, because it’s like a test and we don’t know if that test can hurt [the baby]*” (The Dominican Republic, 28 years old, 12 years in Spain)

“*I don’t know what to tell you, I think not, because if it’s a study… because if it’s a study, it is going to affect the baby… I think not. But, if it’s for everybody’s wellbeing, not affecting my baby, indeed [I would participate]*” (Puerto Arturo, Sucre, 43 years old)

Healthcare professionals explained that they hesitated about women’s willingness to participate in clinical trials. They were concerned that there will be problems regarding acceptability for safety concerns or fear of the vaccine causing harm to babies, but also due to a low perception of the risks for those who have not suffered directly the consequences of ZIKV. They also highlighted the idea that even healthcare professionals would hesitate about recommending pregnant women participation in a clinical trial for possible adverse effects of the vaccine and unknown efficacy for their patients.

“*If you do a clinical trial, it is because its safety or its efficacy is not really clear so… this uncertainty… mmm… would make participants hesitate to accept. Even to healthcare professionals, don’t they? They are the ones that have to believe [in so] before explaining it. So, I think that first it has to be… really confirmed that… so… isn’t it? Like, in previous phases of… of clinical trials, that is safe during pregnancy. And… I don’t know which acceptability would it have, sincerely…*” (Healthcare professional, number 3)

### 3.8. Key Highlights

- Most women were willing to be vaccinated with a hypothetical Zika vaccine;
- However, some women expressed concerns of being vaccinated during pregnancy, yet they would accept it if the vaccine was recommended by a healthcare professional they trust;
- Women would not accept to receive a Zika vaccine while breastfeeding due to fear that their babies may be infected (as opposed to passing their antibodies to them);
- Women reported being more worried about the effects on their children´s health than on themselves when considering receiving a ZIKV vaccine;
- Route administration would not affect women’s decision to accept vaccination. Women perceived injections to act “*more directly, and effectively in not missing a dose*”;
- Some women agreed that the whole population should be vaccinated, not only pregnant women;
- Participants declared that they would not like to participate in a ZIKV vaccine trial due to fear regarding being “*the first ones being tested for a vaccine*” and concerns regarding vaccine safety and its effect on child´s health.

## 4. Discussion

In the current situation, where there is no specific treatment against ZIKV disease, vaccination seems the most promising intervention to prevent the dramatic consequences of infection on pregnant women and their infants. This study aimed at understanding factors affecting vaccine acceptability in our study population as an important step to help develop targeted information and vaccine promotion interventions. In an era of growing vaccine hesitancy and where some vaccination promotion campaigns are not succeeding as expected [41], research on effective pro-vaccine messaging is essential. Our findings provide evidence from high-risk female populations that a future ZIKV vaccine, if available, would be acceptable for women at risk in the event that a new ZIKV outbreak will occur. A high level of vaccine acceptance has been related to an increased knowledge of ZIKV-associated risks and severity. To improve ZIKV vaccine acceptance, messages around vaccine recommendation should describe the benefits of avoiding the negative effects of infection in newborns, while accentuating vaccine safety for the fetuses, children and women, and compatibility with breastfeeding. Evidence shows that engagement of healthcare professionals in promotion of vaccination is highly correlated with high vaccine coverage rates [41]. Women and girls of reproductive age in regions affected by ZIKV constitute the population most at risk, and they should be prioritized in all steps of vaccine development, evaluation, licensing, and distribution. On a global scale, limiting the spread of future outbreaks and holding back the infection will ideally entail mass universal vaccination.

In this article, two different populations, living in diverse contexts, are described. Our aim was not to generalize, but to present those realities within a common research question. The background of the participants in this study was varied and complex. In Spain, some women were permanently living in the country of exposure to the virus and migrated to Catalonia just before or during pregnancy. Others had been living in Spain for many years and acquired the infection during a visit to their friends and relatives in their home countries. All women were attended in the Public Health System, and were participating in a Zika surveillance study [7,42,43]. In Colombia, women came from various backgrounds, as they lived in peri-urban or rural areas in different states of Caribbean Colombia, and had different cultural and educational backgrounds. Furthermore, their familiar economies and social support were different and clearly shaped women’s ability to respond to their children’ needs. Our results showed no differences in women’s responses to receive a hypothetical ZIKV vaccine. Women in both contexts declared that their healthcare professional would guide their decision to be vaccinated, and their main concern was related to possible fetal damage.

Previous research has studied willingness to get a hypothetical ZIKV vaccine in different target populations [14,21,28,30,44]. A study conducted in Indonesia found that 80% of patients who visited outpatient departments would accept to receive a ZIKV vaccine [2], contrary to a 20% of acceptability in pregnant women in Greece [14]. Increased knowledge about ZIKV and access to reliable information on the virus were identified as facilitators of willingness to accept a ZIKV vaccine [28,42]. Few studies have explored vaccine acceptability among pregnant women at risk of ZIKV, and results show that acceptability of a ZIKV vaccine was very high [24,25,28]. However, to our knowledge, no qualitative studies existed that explored the circumstances under which they would accept vaccination, nor that assessed acceptability to vaccination among women who were diagnosed with Zika virus infection or who were caregivers of infants with microcephaly. Alholm et al. concluded that almost half of women participating in their survey expressed strong agreement to receive a hypothetical ZIKV vaccine during pregnancy [29].

The health belief model proposes that individual behavior is directly shaped by perceptions of risk. Perceptions of risk and knowledge are linked to the sources of information they come from, and, consequently, people concerned enough about a disease are probably more likely to adopt health behaviors than the average [28]. People’s motivations to use vaccines would be influenced by their judgements about both the vaccine and the outcomes it prevents [45]. One of the most notable findings from this study is that the majority of participants intended to receive a ZIKV vaccine. This may be related to an increased perception of risk and severity as a result of most of our population having gone through infection or living in a ZIKV highly endemic and severely affected area. Another study performed in the USA showed that risk perception and concerns about acquiring ZIKV infection while traveling was found to be the only positive predictor of ZIKV vaccine acceptance, and it was higher among female participants who were planning a pregnancy [31]. On the contrary, knowledge of ZIKV symptoms in adults was a negative predictor of willingness to receive a ZIKV vaccine. This study was conducted in March 2017 when the peak of the epidemic was over, which could have influenced the results [31]. These results are in line with our findings. Risk perception was a facilitator for vaccine acceptance, as was the willingness of having more children in the future. In general, women were not looking for a protective effect for themselves, but for their babies, since they perceived that ZIKV cannot harm non-pregnant people. Participants were very conscious about reducing the risk of CZS or protecting their children’s health in a hypothetical future pregnancy, as these were reported as being the major reasons to accept vaccination.

Studies on the acceptability of vaccines for pregnant women are critical for vaccine investment, demand strategies, effective implementation, messaging strategies and modes of communication are key elements to increase acceptability [46]. Healthcare providers have been identified as key facilitators for vaccine acceptance [29], particularly among women from Central and South America, given the strong vaccine uptake engagement in the region. Frequent misinformation about vaccines in the media make it even more important for healthcare professionals to provide accurate information to their patients in order to increase vaccine acceptability. Studies have shown that when information provided by healthcare professionals is insufficient, people might investigate other sources that may contain misleading information, or information that may be misinterpreted [7,47]. Furthermore, our results show that the willingness to accept this hypothetical vaccination during pregnancy is lower than during other periods of women’s lives. Regarding the preferred route of administration, women trusted injected vaccines more because they believe that injected vaccines act quicker and are more efficacious.

Our results are in line with a recent literature review assessing vaccine hesitancy and barriers for vaccination among pregnant women, where the main concern for accepting vaccination was safety in pregnancy and possible negative consequences on the fetus [41]. In our study, recommendations from healthcare workers were reported to increase positive attitudes about vaccine effectiveness and safety, and, as a consequence, to increase vaccination uptake. In a study in the US, high willingness to participate in a ZIKV vaccine trial in pregnant women was associated with the trust on safety evidence communicated by healthcare professionals [24]. However, in our study, women were less likely to accept participation in a clinical trial primarily due to safety concerns, but among those who suffered directly the consequences of Zika infection and declined participating in a trial, some declared they would reconsider it if by doing so they could help other women to avoid the negative experience they went through. Differences among Goldfarb et al. [24] and our study could be explained as our objectives, design, methodology and population under study are very different. Harapan et al. [23] explained how financial compensation might play a role in the inclusion of participants in clinical trials, but it is not a legal recommendation to boost participation in clinical trials in certain countries, such as Spain. The study of Harapan et al. [23] also highlighted the importance of participants’ knowledge about ZIKV and attitudes towards childhood vaccination as determining factors to participate in a vaccine trial. The Ethics Working Group on ZIKV Research & Pregnancy provided a series of recommendations to provide guidance in addressing the needs of pregnant women once a ZIKV vaccine is available: to develop a vaccine that can be responsibly and effectively used during pregnancy, to collect data specific to safety and immunogenicity in pregnancy, and to ensure pregnant women have fair access to participate in Zika virus vaccine trials [48]. This last imperative was highlighted because of the exclusion of pregnant and breastfeeding women from clinical trials for the Ebola vaccine [48].

Another identified barrier to vaccination among our study population was the feeling of worry about needing to stop breastfeeding their babies because of the vaccine. Vaccines developed for maternal immunization often aim to provide passive immunity to the baby through the transfer of antibodies through breast milk [49]. Participants’ concerns were associated with a misbelief about transmitting the virus to the infant through breastfeeding as opposed to passing their antibodies to them. Similar concerns about influenza vaccine safety during breastfeeding have also been reported [50]. Therefore, it is a subject of special consideration for vaccine delivery in this population.

Significantly, some participants agreed on the fact that, if available, a ZIKV vaccine should be administered to the whole population, regardless of sex or age. Specifically, this was proposed by women who were previously aware of the sexual transmission of the virus. While the 2015 epidemic proved that women bear the highest burden, it is undeniable that the consequences of a congenital Zika infection have repercussions on the whole family unit. The WHO placed the focus on women of reproductive age as a target group for ZIKV immunization, scaling it up to men and male adolescents if this would become possible in the future. Similar discussions have been taking place in relation to the human papillomavirus (HPV) vaccine. While in the beginning this vaccine targeted adolescent girls, a few countries have recently started to vaccinate young boys with the intention of protecting them from anal and oral cancer [51]. This strategy is also considered to effectively achieve herd immunity through a rapid decline in the viral load among the population, reducing the incidence of sexually acquired HPV among both men and women. In the case of ZIKV, where vector transmission does not discern between sex or gender, mass vaccination would probably have a similar impact in reducing viral loads and transmission in the population. Finally, another interesting finding is that only one participant in our study spoke about the cost of a hypothetical ZIKV vaccine as a barrier or facilitator to get the immunization. As this was not a “willingness to pay” study, this topic was not directly investigated during the interviews, but it is an interesting one to explore in future research.

An issue that might be interpreted as a limitation to this study is the total number of women that participated in the study; however, the authors did not perceive this as a concern, as the saturation point was reached at the time of the analysis. The study had strengths, such as the fact that insights around ZIKV were obtained by women who were affected directly by ZIKV, and who faced the challenges of ZIKV from the beginning of the epidemic in 2015 in different ways. Their views provide us with an excellent image that could influence current health campaigns and communication strategies. However, qualitative results need to be interpreted with caution, as generalization cannot be performed. A strength of the study is that all interviewers and all study staff spoke the same language as study participants (Spanish). The benefits of speaking the same language and interviewers’ experience might have translated into increased cooperation from participants who seemed comfortable, were willing to share their experiences and participated mindfully in in-depth discussions.

While ZIKV vaccines are still in clinical development, there is scarce literature addressing acceptance of a hypothetical vaccine by populations at risk and among affected women. Our results represent an important contribution to the literature, providing evidence of ZIKV vaccine acceptability and participation in ZIKV vaccine trials among women that have experienced ZIKV infection during pregnancy, specifically migrant women living in an European context where there is no local transmission, and among mothers of children with microcephaly living in a country heavily affected by the ZIKV epidemic where endemic transmission still occurs. Thus, this study represents an important contribution by trying to understand if such perceptions and other demographic factors may predict individual’s acceptance of a promising future ZIKV vaccine.

## 5. Conclusions

Results show a high acceptability of a potential ZIKV vaccine indicated by willingness to be vaccinated among women who had a previous experience with ZIKV. That acceptance is related to the level of knowledge about ZIKV, associated risks and severity. The study highlights that for ZIKV vaccine acceptance, messages during vaccine delivery need to address the benefits associated with vaccination and the risks avoided, while emphasizing the safety of the vaccine for the fetus and infant, and compatibility with breastfeeding. Lastly, the engagement of healthcare professionals for effective information and communication about potential ZIKV vaccines is a key element for successful implementation.

Women and girls of reproductive age in regions affected by ZIKV comprise the population that could most benefit from introduction of ZIKV vaccines, and, thus, they should be prioritized in all steps from vaccine development to evaluation and implementation. On a global scale, limiting the spread of future outbreaks and holding back the infection will ideally entail mass universal vaccination.

## Figures and Tables

**Table 1:** Sociodemographic characteristics of women participating in the study.

Characteristics	In Spain (*n* = 17)	In Colombia (*n* = 8)
	**n (%)**	**n (%)**
**Age (years)**		
<20	-	1 (13)
20–25	3 (18)	4 (50)
26–30	2 (12)	1 (13)
31–35	10 (59)	1 (13)
>35	2 (12)	1 ** (13)
**Nationality**		
Dominican	6 (35)	-
Honduran	5 (29)	-
Colombian	2 (12)	8 (100)
Venezuelan	2 (12)	-
Guatemalan	1 (6)	-
Brazilian	1 (6)	-
**Education**		
Primary	1 (7)	2 (25)
Secondary	8 (53)	3 (38)
University or higher	6 * (40)	3 (38)
**Occupation**		
No own income	10 (59)	4 (50)
Formal remuneration	7 (41)	4 (50)
**Civil status**		
Married/living with partner	15 (88)	8 (100)
Single	2 (12)	-
**Socioeconomic status**		
Low	Not applicable	8 *** (100)

* Missing values (2 for Education). ** One woman (43 years old) was the grandmother but the primary caregiver. *** The official strata division in Colombia refers to strata 1, 2 and 3 as “low socioeconomic status”; strata 4 is considered “lower-middle”; and strata 5 and 6 “high socioeconomic status”.

**Table 2:** Reasons for accepting a hypothetical Zika virus (ZIKV) vaccine.

Reasons to Accept a ZIKV Vaccine	Quotes from Participants in Catalunya, Spain	Quotes from Participants in Caribbean Colombia
To protect children’s health	“*To take care of the baby, and also to take care of myself, but mainly to take care of the baby*” (The Dominican Republic, 32 years old, 18 years in Spain)	“*Because one never knows if oneself is prone another time this [infection in the baby] to happen*” (Montería, Córdoba, 18 years old)
To avoid disease and health problems in the future	“*If there’s a vaccine, it would avoid a lot of things. It would avoid a lot of cases, won’t it? Because… I think that if there’s a vaccine, people are going to look for it, to get vaccinated, to avoid this kind of things*” (Brazil, 36 years old, 11 years in Spain)	“*I would not like to be with that risk that I had, [I] could have more problems in the future*” (Cartagena, Bolívar, 22 years old)
To deal with negative state of mind/maintain mental health	“*In order to be more relaxed if there’s the virus or not, in general. Because I know I am going to be ‘sheltered’ [protected]” (Venezuela, 25 years old, 1 year in Spain)“Not to live the uncertainty I lived before*” (The Dominican Republic, 34 years old, 15 years in Spain)	“*To avoid something as it happened to me […] In my pregnancy I had fear*” (Sincelejo, Sucre, 20 years old)
To avoid ZIKV infection	“*To prevent disease, if there’s an epidemic… and this vaccine is given, I mean, that could have prevented me from having the disease, I would do so [get it].*” (Honduras, 22 years old, 3 years in Spain)	“*Without thinking about it [being vaccinated] twice, if it [vaccine] existed, if those risks exist and vaccine exists, to control all those viruses, as they [vaccines] exist to control others, without doubting*” (Magangué, Bolívar, Colombia 29 years old)
Trust on healthcare professionals	“*Yes. Well, depends on what the doctor says. Yes… […] Because there are many drugs that one cannot take being pregnant, such as breastfeeding… So, it depends on what the doctor says*” (Venezuela, 25 years old, 1 year in Spain)	“*If the doctor recommends me to get vaccinated, indeed!*” (Sincelejo, Sucre, 24 years old)
Experience of already having a sick child due to ZIKV	Not applicable	“*As we already passed through this process… it’s like… it marks one deeply, and the decision [to be vaccinated] it’s, as first sight!*” (Magangué, Bolívar, Colombia 29 years old)
As a moral personal obligation	“*To prevent disease, because now it is my obligation to do so*” (Honduras, 22 years old, 3 years in Spain)	Nothing similar reported

## References

[1] Wikan N., Smith D.R. (2016). Zika virus: History of a newly emerging arbovirus. Lancet Infect. Dis..

[2] Luo X.S., Imai N., Dorigatti I. (2020). Quantifying the risk of Zika virus spread in Asia during the 2015–16 epidemic in Latin America and the Caribbean: A modeling study. Travel Med. Infect. Dis..

[3] Krauer F., Riesen M., Reveiz L., Oladapo O.T., Martinez-Vega R., Porgo T.V., Haefliger A., Broutet N.J., Low N., WHO Zika Causality Working Group (2017). Zika Virus Infection as a Cause of Congenital Brain Abnormalities and Guillain-Barre Syndrome: Systematic Review. PLoS Med..

[4] Faical A.V., de Oliveira J.C., Oliveira J.V.V., de Almeida B.L., Agra I.A., Alcantara L.C.J., Acosta A.X., de Siqueira I.C. (2019). Neurodevelopmental delay in normocephalic children with in utero exposure to Zika virus. BMJ Paediatr. Open.

[5] Mulkey S.B., Arroyave-Wessel M., Peyton C., Bulas D.I., Fourzali Y., Jiang J., Russo S., McCarter R., Msall M.E., Du Plessis A.J. (2020). Neurodevelopmental Abnormalities in Children With In Utero Zika Virus Exposure Without Congenital Zika Syndrome. JAMA Pediatr..

[6] Kelly A.H., Lezaun J., Löwy I., Matta G.C., de Oliveira Nogueira C., Rabello E.T. (2020). Uncertainty in times of medical emergency: Knowledge gaps and structural ignorance during the Brazilian Zika crisis. Soc. Sci. Med..

[7] Marbán-Castro E., Villén-Gonzalvo A., Enguita-Fernàndez C., Marín-Cos A., Menéndez C., Maixenchs M., Bardají A. (2020). Uncertainties, Fear and Stigma: Perceptions of Zika Virus among Pregnant Women in Spain. Int. J. Environ. Res. Public Health.

[8] Stellmach D., Beshar I., Bedford J., du Cros P., Stringer B. (2018). Anthropology in public health emergencies: What is anthropology good for?. BMJ Glob. Health.

[9] Hewlett B.S., Epelboin A., Hewlett B.L., Formenty P. (2005). Medical anthropology and Ebola in Congo: Cultural models and humanistic care. Bull. Soc. Pathol. Exot..

[10] Brown H., Kelly A.H., Mari Saez A., Fichet-Calvet E., Ansumana R., Bonwitt J., Magassouba N.F., Sahr F., Borchert M. (2015). Extending the “social”: Anthropological contributions to the study of viral haemorrhagic fevers. PLoS Negl. Trop. Dis..

[11] MacGregor H. (2020). Novelty and Uncertainty: Social Science Contributions to a Response to COVID-19. http://somatosphere.net/forumpost/novelty-and-uncertainty/.

[12] Brown P. (2020). Studying COVID-19 in light of critical approaches to risk and uncertainty: Research pathways, conceptual tools, and some magic from Mary Douglas. Health Risk Soc..

[13] Passos M.J., Matta G., Lyra T.M., Moreira M.E.L., Kuper H., Penn-Kekana L., Mendonça M. (2020). The promise and pitfalls of social science research in an emergency: Lessons from studying the Zika epidemic in Brazil, 2015–2016. BMJ Glob. Health.

[14] Mouchtouri V.A., Papagiannis D., Katsioulis A., Rachiotis G., Dafopoulos K., Hadjichristodoulou C. (2017). Knowledge, Attitudes, and Practices about the Prevention of Mosquito Bites and Zika Virus Disease in Pregnant Women in Greece. Int. J. Environ. Res. Public Health.

[15] Painter J.E., Plaster A.N., Tjersland D.H., Jacobsen K.H. (2017). Zika virus knowledge, attitudes, and vaccine interest among university students. Vaccine.

[16] Maslow J.N. (2019). Zika Vaccine Development-Current Progress and Challenges for the Future. Trop. Med. Infect. Dis..

[17] Garg H., Mehmetoglu-Gurbuz T., Joshi A. (2018). Recent Advances in Zika Virus Vaccines. Viruses.

[18] WHO Vaccine Pipeline Tracker. https://www.who.int/immunization/research/vaccine_pipeline_tracker_spreadsheet/en/.

[19] Poland G.A., Ovsyannikova I.G., Kennedy R.B. (2019). Zika Vaccine Development: Current Status. Mayo Clin. Proc..

[20] Guedes G.R., Coutinho R.Z., Marteleto L., Pereira W.H.S., Duarte D. (2018). Why social perception matters during disease outbreaks: Looking at how individuals understand the Zika virus by self-reported history of infection. Cadernos de Saude Publica Brazil.

[21] Fatima K., Syed N.I. (2018). Dengvaxia controversy: Impact on vaccine hesitancy. J. Glob. Health.

[22] Dredze M., Broniatowski D.A., Hilyard K.M. (2016). Zika vaccine misconceptions: A social media analysis. Vaccine.

[23] Harapan H., Mudatsir M., Yufika A., Nawawi Y., Wahyuniati N., Anwar S., Yusri F., Haryanti N., Wijayanti N.P., Rizal R. (2019). Community acceptance and willingness-to-pay for a hypothetical Zika vaccine: A cross-sectional study in Indonesia. Vaccine.

[24] Goldfarb I.T., Jaffe E., James K., Lyerly A.D. (2018). Pregnant women’s attitudes toward Zika virus vaccine trial participation. Vaccine.

[25] Fraiz L.D., de Roche A., Mauro C., Catallozzi M., Zimet G.D., Shapiro G.K., Rosenthal S.L.U.S. (2018). pregnant women’s knowledge and attitudes about behavioral strategies and vaccines to prevent Zika acquisition. Vaccine.

[26] Harapan H., Mudatsir M., Yufika A., Nawawi Y., Wahyuniati N., Anwar S., Yusri F., Haryanti N., Wijayanti N.P., Rizal R. (2018). Willingness to Participate and Associated Factors in a Zika Vaccine Trial in Indonesia: A Cross-Sectional Study. Viruses.

[27] Muniz R.L., Godoi I.P., Reis E.A., Garcia M.M., Guerra-Junior A.A., Godman B., Ruas C.M. (2019). Consumer willingness to pay for a hypothetical Zika vaccine in Brazil and the implications. Expert Rev. Pharmacoecon. Outcomes Res..

[28] Wong L.P., Alias H., Hassan J., AbuBakar S. (2017). Attitudes towards Zika screening and vaccination acceptability among pregnant women in Malaysia. Vaccine.

[29] Alholm Z., Ault K., Zwick R., Fitzgerald S., Satterwhite C. (2017). Pregnant Women’s Acceptance of Hypothetical Zika Vaccine. Open Forum Infect. Dis..

[30] Olson D., Rick A.-M., Krager S., Lamb M., Asturias E.J. (2018). Vaccine Demand and Willingness-to-pay for Arbovirus Vaccines: A Cross-sectional Survey in Rural Guatemala. Pediatr. Infect. Dis. J..

[31] Vielot N.A., Stamm L., Herrington J., Squiers L., Kelly B., McCormack L., Becker-Dreps S. (2018). United States Travelers’ Concern about Zika Infection and Willingness to Receive a Hypothetical Zika Vaccine. Am. J. Trop. Med. Hyg..

[32] Mendez N., Oviedo-Pastrana M., Mattar S., Caicedo-Castro I., Arrieta G. (2017). Zika virus disease, microcephaly and Guillain-Barre syndrome in Colombia: Epidemiological situation during 21 months of the Zika virus outbreak, 2015–2017. Arch. Public Health.

[33] Mattar S., Ojeda C., Arboleda J., Arrieta G., Bosch I., Botia I., Alvis-Guzman N., Perez-Yepes C., Gerhke L., Montero G. (2017). Case report: Microcephaly associated with Zika virus infection, Colombia. BMC Infect. Dis..

[34] Strauss A., Corbin J. (1990). Basics of Qualitative Research: Grounded Theory Procedures and Techniques.

[35] Bowen G.A. (2008). Naturalistic inquiry and the saturation concept: A research note. Qual. Res..

[36] Glaser B., Strauss A. (1967). The Discovery of Grounded Theory: Strategies for Qualitative Research.

[37] Thyer B., Holosko M.J. (2016). Overview of Qualitative Research Methods. Handb. Soc. Work Res. Methods.

[38] Ritchie J., Lewis H. (2003). Qualitative Research Practice: A Guide for Social Science Students and Researchers.

[39] Tong A., Sainsbury P., Craig J. (2007). Consolidated criteria for reporting qualitative research (COREQ): A 32- item checklist for interviews and focus group. Int. J. Qual. Health Care.

[40] McHale T.C., Romero-Vivas C.M., Fronterre C., Arango-Padilla P., Waterlow N.R., Nix C.D., Falconar A.K., Cano J. (2019). Spatiotemporal Heterogeneity in the Distribution of Chikungunya and Zika Virus Case Incidences during their 2014 to 2016 Epidemics in Barranquilla, Colombia. Int. J. Environ. Res. Public Health.

[41] Wilson R.J., Paterson P., Jarrett C., Larson H.J. (2015). Understanding factors influencing vaccination acceptance during pregnancy globally: A literature review. Vaccine.

[42] Goncé A., Martinez M.J., Marbán-Castro E., Saco A., Soler A., Alvarez-Mora M.I., Peiro A., Gonzalo V., Hale G., Bhatnagar J. (2018). Spontaneous Abortion Associated with Zika Virus Infection and Persistent Viremia. Emerg. Infect. Dis. United States.

[43] Sulleiro E., Rando A., Alejo-Cancho I., Bardají A., Fumadó V., Soriano Arandes A., Muñoz J., Martínez A., Jané M., Marbán-Castro E. (2019). Screening for Zika virus infection in 1057 potentially exposed pregnant women, Catalonia (northeastern Spain). Travel Med. Infect. Dis. Netherlands.

[44] Patel N., Anees M., Kola R., Acuña J., Rodriguez P., Vega D. (2019). Association between Knowledge of Zika Transmission and Preventative Measures among Latinas of Childbearing Age in Farm-Working Communities in South Florida. Int. J. Environ. Res. Public Health.

[45] Ophir Y., Jamieson K.H. (2018). Intentions to use a novel Zika vaccine: The effects of misbeliefs about the MMR vaccine and perceptions about Zika. J. Public Health (Oxf.).

[46] PATH (2018). Advancing RSV Maternal Immunization: A Gap Analysis Report. https://path.azureedge.net/media/documents/Advancing_RSV_Maternal_Immunization__A_Gap_Analysis_Report.pdf.

[47] Amith M., Cunningham R., Savas L.S., Boom J., Schvaneveldt R., Tao C., Cohen T. (2017). Using Pathfinder networks to discover alignment between expert and consumer conceptual knowledge from online vaccine content. J. Biomed. Inform..

[48] Schwartz D.A. (2018). Clinical Trials and Administration of Zika Virus Vaccine in Pregnant Women: Lessons (that Should Have Been) Learned from Excluding Immunization with the Ebola Vaccine during Pregnancy and Lactation. Vaccines..

[49] Faucette A.N., Pawlitz M.D., Pei B., Yao F., Chen K. (2015). Immunization of pregnant women: Future of early infant protection. Hum. Vaccines Immunother..

[50] Lynch M.M., Mitchell E.W., Williams J.L., Brumbaugh K., Jones-Bell M., Pinkney D.E., Layton C.M., Mersereau P.W., Kendrick J.S., Medina P.E. (2012). Pregnant and recently pregnant women’s perceptions about influenza a pandemic (H1N1) 2009: Implications for public health and provider communication. Matern. Child Health J..

[51] Stanley M. (2014). HPV vaccination in boys and men. Hum. Vaccines Immunother..

